# Antecedent Factors of Green Purchasing Behavior: Learning Experiences, Social Cognitive Factors, and Green Marketing

**DOI:** 10.3389/fpsyg.2021.777531

**Published:** 2021-12-08

**Authors:** Aries Susanty, Nia Budi Puspitasari, Heru Prastawa, Pradhipta Listyawardhani, Benny Tjahjono

**Affiliations:** ^1^Department of Industrial Engineering, Diponegoro University, Semarang, Indonesia; ^2^Centre for Business in Society, Coventry University, Coventry, United Kingdom

**Keywords:** learning experiences, cognitive factors, green personal care product, purchase intention, purchase behavior

## Abstract

This study extends the theory of planned behavior (TPB) framework by introducing three further variables (i.e., learning experience, contextual factors, and green marketing) to explain how behavioral intention and actual behavior are induced by situational factors as well as green advertising from the company. Then, this study has four objectives. First, this study will assess the direct effect of personal factors (i.e., demographic factors) and contextual factors on learning experience and the direct effect of personal factors (i.e., demographic factors) on subjective norms. Second, this study will assess the direct effect of learning experience on social cognitive factors for a specific green product. Third, this study will assess the direct effect of social cognitive factors on intention purchasing behavior. Finally, this study will assess the role of green marketing as a moderating variable for the relationship between intention and purchasing behavior. Data used in this study were primary data, which were collected through closed questionnaires with a five-point Likert scale. This study succeeded in obtaining 602 valid data from the results of filling out questionnaires by participants. This study uses the partial least square (PLS) method with SmartPLS 3.0 for data processing. The result of data processing indicated that outcome expectation, self-efficacy, and subjective norms had significant positive effects on purchase intention for green personal care products. This study also found that the learning experience influenced both outcome expectation and self-efficacy. Although weak, the learning experience was influenced by demographic factors and contextual factors. The demographic factors that affect learning experience were gender and level of education. The contextual factor has a more substantial influence on learning experience in developing consumption-related attitudes to green personal care products than the demographic factor. Finally, this study also found the direct effect of intention on actual purchase behavior and the positive role of green marketing as the moderating variable.

## Introduction

Marketers have been talking about green consumption since the 1960s (Rahman et al., 2017). In the recent decade, the concept and application of green consumption as well as environmental responsibility behavior have become an essential issue in the literature of marketing (Leonidou et al., 2013; Peloza et al., 2013; Haws et al., 2014) as the environmental consciousness and the attitude of customers toward the environment indicate a positive trend [see the result of the previous study from CEAP (2007), Eurobarometer (2011), and Nielsen (2014)]. However, although the environmental consciousness and attitude of customers toward the environment indicate a positive trend, empirical evidence indicates that attitude infrequently translates into actual purchase behavior. It means there is a discrepancy or “gap” between consumers articulated favorable attitudes and actual purchasing practices (e.g., Vermeir and Verbeke, 2008; Zabkar and Hosta, 2012; Gleim et al., 2013).

The evidence for the inconsistency has been recorded in different countries (Nguyen et al., 2019), making the scholars frequently called for further research to close the inconsistency. If we can determine the antecedent variables through rigorous study, valuable steps and strategies can be taken to reduce the inconsistency and encourage consumers to purchase green products. Then, many conceptual theories have been developed to explain the reason for green purchasing behavior, which starts with the green intention first, such as the theory of reasoned action (TRA) (Ajzen and Fishbein, 1980), and its extension, the theory of planned behavior (Ajzen, 1991), general theory of marketing ethics (Hunt and Vitell, 1986), norm activation model and value-belief-norm theory (Stern, 1999), construal level theory (Trope and Liberman, 2003), and social practice approach and social cognitive theory (Bandura, 2008; Shove and Walker, 2010). Among all these theories, the most widely used was the TRA and its extension, the TPB (Ceglia et al., 2015; Hanss et al., 2016). However, although widely used, TPB has some limitations that cause the researchers to propose various extended forms of TPB. For example, Chen and Hung (2016) extended TPB by including environmental consciousness, social impression, and environmental ethics and beliefs into its framework. Sreen et al. (2018) extended TPB by including long-term orientation, collectivism, and man-nature orientation factors into its framework.

Previous studies have used and extended TPB by including several antecedent variables into this framework to explore the purchasing behavior of green products. However, there are still many limitations that should be solved (Zhang et al., 2019). One of the limitations is related to the hypothesis of the TPB framework. According to the framework, people were hypothesized as a homogeneous individual who get behavioral intention decision or even behavioral purchasing decision only based on three variables, namely, attitude, subjective norm, and perceived behavioral control; the factors of differences in individuals, culture, and contexts are excluded from the TPB framework (Zhang, 2018). These limitations encourage more exploration of the implementation of the TPB framework in exploring green purchasing behavior. So, this present study tried to extend the TPB by including social cognitive theory (SCT), social learning theory (SLT), and green marketing in its framework within the limitation of TPB. The cognitive view holds that people are not homogeneous, and the behavior of people is based on information-seeking and is usually directed by a specific goal (Liu et al., 2018). Then, the social cognitive theory focuses on how behaviors are influenced by observing others and how these observations shape social behaviors and cognitive processes (Bandura, 1986). Bandura (1986) proposed that these three interacting variables, namely, personal factors, environment, and behavior, might explain human actions. Furthermore, it is suggested in social learning theory that individuals adopt general behaviors and attitudes through seeing other people or by observing electronic or print media (Martin and Bush, 2000). Then, the concept of green marketing will influence the efficiency of the cognitive persuasion strategies (Hartmann and Apaolaza, 2006), in which many earlier study has demonstrated the beneficial impact of green marketing on customer attitudes toward green purchasing (e.g., Lang and Hyde, 2013; Kotler et al., 2014). Shortly, in this study, the factors belonging to SCT, SLT, and green marketing were used as an antecedent variable to measure its effect on the actual purchasing behavior of green products through purchase intention. Then, the green marketing factor was used as a moderating variable to increase purchase intention to actual purchasing behavior. There are, hence, four objectives in this study in detail.

1. This study will assess the direct effect of personal factors (i.e., demographic factors) and contextual factors on learning experience and the direct effect of personal factors (i.e., demographic factors) on subjective norms.
2. This study will assess the direct effect of learning experience on social cognitive factors for a specific green product.
3. This study will assess the direct effect of social cognitive factors on intention purchasing behavior.
4. This study will assess the role of green marketing as a moderating variable for the relationship between intention and purchasing behavior.

The product that becomes the subject of the study is green personal care or green toiletries product. Personal care products are a source of concern for the environment since their components have been found in all water bodies worldwide. Moreover, there is fewer green personal care product compared with general personal care. So, since personal care products have already become one of the essential needs of the people and they are covering a wide range of categories (such as hair care, skincare, baby care, oral care, etc.) as well as being produced by different manufacturing companies, the result of this study can be used as the input for manufacturers to consider the significant cognitive variable that could drive the customer purchasing behavior of green personal care product in their marketing strategy. It is not impossible since our previous study indicated that people have a high tendency for shifting to environmentally friendly personal care products (Susanty et al., 2021).

## Literature Review

### Theory of Planned Behavior

The theory of planned behavior (TPB) is the work of Ajzen (1991). Three constructs are used to determine the behavior of a person in this theory, namely, attitude toward behavior, perceived behavioral control, and subjective norms. Many studies have used and extended the TPB, including those which consider green customer behavior from a psychological perspective to understand the influence of those three constructs on product purchase intentions (such as research conducted by Kun-Shan and Yi-Man, 2011; Paul et al., 2016; Yadav and Pathak, 2017; Liu et al., 2018; Ting et al., 2019; Yarimoglu and Gunay, 2020).

The first construct in the TPB is the attitude toward behavior. The attitude toward a behavior is defined by Ajzen (1991) as either a positive or negative assessment of that behavior. Perceived behavioral control is the second construct in the TPB. Ajzen and Madden (1986) defined this construct as the perceived complexity of an action. The perception of behavioral control is determined by trust in opportunities and resources. Lastly, there is the subjective norm, which is the third construct of the TPB. Ajzen (1991) and O’Neal (2007) defined it as societal pressure to take part in or refrain from participating in a particular activity.

### Social Cognitive Theory

Bandura is the first researcher who introduces the social cognitive theory (SCT) (Bandura, 1986, 2006). Critical to SCT are the concepts of outcome expectations and self-efficacy (Bandura, 1986). Outcome expectation can be described as the result that a person expects to achieve by performing a particular action. Individuals will engage in these behaviors if they feel the consequences will be beneficial (Lin and Hsu, 2015). The concept of outcome expectation in SCT is synonymous with a term for an attitude toward behavior in the TPB context, since both interpreted an outcome as a result of an act rather than the act itself (Autio et al., 2010).

Self-efficacy is described as belief of an individual on his/her ability to complete a task with specific skills rather than his/her ability to do so. It is based not on his/her ability but on his/her belief in what one can do with those abilities (Bandura, 1986). Individuals with high self-efficacy will expect positive results, while those with low self-efficacy will expect average or even poor results (Bandura, 1986). In the TPB framework, self-efficacy in SCT is synonymous with perceived behavioral control, although some researchers see a slight difference between self-efficacy and perceived behavioral control. Perceived behavioral control focuses more on the perceived ability to perform a behavior, whereas self-efficacy strongly focuses more on the perceived capability to bring about the desired outcome (Hanss and Böhm, 2010). Without ignoring this slight difference, in the recent variants of TPB, Fishbein and Cappella (2006) have relabeled perceived behavioral control to self-efficacy. Then, in terms of green buying behavior, the self-efficacy of green customers can reflect their mindset that they have the potential or capability to identify and buy environmentally friendly products (Preko, 2017).

### Outcome Expectation, Self-Efficacy, and Subjective Norms on Green Purchase Intention

Expected positive and negative physical activity effects are used in the SCT to conceptualize the outcome expectation. Other outcome expectation hypotheses, such as subjective expected utility theory and behavioral economics theory, claim that the choice of individuals to respond in a specific manner is based on their expected outcomes of potential behavioral alternatives (Williams et al., 2005). Then, based on these conditions, several previous studies have shown the role of outcome expectation as an indicator of green purchase intention, such as Lin and Hsu, 2015; Liou et al., 2019). Lin and Hsu (2015) found that the outcome expectation of an individual is linked to his/her green purchasing actions. In this scenario, positive benefits (e.g., compensation or a feeling of pride in helping the environment) will improve the motivation and ability of an individual to engage in green consumption. Liou et al. (2019) showed that the higher the “outcome expectation of air pollution control and prevention” of an individual is, the higher the extent of the “willingness to participate in air pollution control and prevention” of an individual will be. Briefly, since outcome expectation is a belief of the consequences resulting from behavior and a judgment before action, the first hypothesis in the context of green personal care was proposed.

H1: Outcome expectation is positively affecting the purchase intention for green personal care product; outcome expectation of buying green personal care product related to the belief of the positive consequences resulting from a behavior of buying green personal care product.

Self-efficacy is one of the cognitive factors that believed to have an essential role in prosocial or proenvironmental behaviors (Hanss and Böhm, 2010; Tagkaloglou and Kasser, 2018; Oliver et al., 2019), which further can lead to green purchasing intention. In this case, the positive relationship between self-efficacy and green purchasing intention can be seen in previous studies conducted by Sharma and Dayal (2016), Han and Hyun (2017). Sharma and Dayal (2016) discovered a direct and indirect positive relationship between self-efficacy and green purchasing intentions of consumers. Green purchasing intention will be higher when beliefs of consumers lead to their conscious action to minimize the negative impact on the environment if they are efficacious. Han and Hyun (2017) also found that self-efficacy has a positive and significant impact on the proenvironmental intentions of museum visitors. So, based on the previous research, it is clear that self-efficacy can be used to predict green purchasing intention. As a result, the second hypothesis in the context of green personal care was proposed.

H2: Self-efficacy is positively affecting the purchase intention for green personal care product; self-efficacy related to believe that he/she will lead to their conscious action to minimize the negative impact on the environment if they buy green personal care product.

There was no agreement about how subjective norms influence green product purchasing intention. Although some previous researchers found that subjective norms have a negative effect on green product purchasing intention (Lee, 2010), the majority of recent studies have looked at the positive effect of subjective norms on green product purchasing intention (Moons and De Pelsmacker, 2012; Wu and Chen, 2014; Yazdanpanah and Forouzani, 2015; Sreen et al., 2018; Zhang et al., 2019). For example, a study on electric car usage found a significant relationship between subjective norms and electric car usage (Moons and De Pelsmacker, 2012). Zhang et al. (2019) discovered the positive effect of subjective norms on purchase intention for energy-efficient household appliances and purchase intention for organic clothing. Then, the third hypothesis was developed based on the research mentioned above.

H3: Subjective norms are positively affecting the purchase intention for a green personal care product.

### Learning Experience, Outcome Expectation, and Self-Efficacy

The presence of learning experience in the proposed conceptual model can be traced back to the explanation of Bandura. Within SLT, individuals acquire general behaviors and attitudes by copying the actions or previous experiences of other people (Martin and Bush, 2000). Individual consumers also acquire consumption-related attitudes and behaviors as a result of their learning experiences. These experiences may occur in a number of situations when customers are exposed to a variety of diverse influences and adventures, and they are especially important in shaping the customer behavior of young adults and teens (Martin and Bush, 2000). Learning is largely a knowledge-processing technique in SCT. Information regarding behavior structure and environmental events is transformed into symbolic representations that serve as action guides (Bandura, 1986). As a result, since behavior connected to outcome expectation (belief in good outcomes as a result of an action) and self-efficacy (capacity of an individual to execute) may be developed *via* learning experiences, this study proposed the fourth and fifth hypothesis.

H4: Learning experiences in developing consumption-related attitudes are positively affecting the outcome expectation.

H5: Learning experiences in developing consumption-related attitudes are positively affecting the self-efficacy.

### Contextual and Demographic Factors

The contextual factor denotes an external condition that affects the behavior of customers. Contextual factors, such as standard of quality, characteristics of the product, availability of recycling facilities, the market supply of materials, physical infrastructure, and policy incentives, can influence individual environmental behavior, which in turn will influence intention to purchase the green product (Santos, 2008; Zepeda and Deal, 2009). Zepeda and Deal (2009) discovered that the contextual factors could be seen as an incentive for buying behavior. It does not solely depend on general motivation as the contextual factor impacts individual motivation too. For example, even if a person is interested in purchasing green products, they cannot purchase such a product if they are not presented for sale in a reachable place (Tanner and Wölfing Kast, 2003).

Additionally, this study extends the construct by employing learning experiences factors on the impact of contextual factors on customer green purchasing behavior, since Astin (1984) and Vondracek et al. (1986) highlight that contextual factor (resources, opportunities, affordances, or barriers) presented by a particular environmental variable may be subject to individual interpretation. Thus, it may encourage or inhibit the willingness of learner to take responsibility for his/her learning. Based on the research as mentioned above, the sixth hypotheses were proposed in this study.

H6: Contextual factors are positively affecting the learning experiences in developing consumption-related attitudes.

Scholars have investigated the differences in learning approach/process/result based on demographic factors (i.e., sex, age, level of education, and level income), among other Remali et al. (2013), Xie and Zhang (2015), Aristovnik et al. (2017), Radhika and Nivedha (2020), and others. Although the result seemed inconsistent, on average, they found the significant effect of different demographic factors on the learning approach/process/result. Additionally, this study extends the research of the effect of demographic factors on learning by investigating its effect on the learning experience in developing consumption-related attitudes and behaviors. Then, the seventh hypothesis was proposed in this study.

H7: Demographic factors are positively affecting the learning experiences in developing consumption-related attitudes.

Moreover, demographic factors (i.e., gender, age, level of education, and level of income) have a different effect on green purchasing behavior (Rizwan et al., 2013; Du et al., 2018; Shao et al., 2018; Wang et al., 2018; Li et al., 2019; Song et al., 2019; Zhang et al., 2019), as well as on subjective norms (Venkatesh and Morris, 2000; Morris et al., 2005; White Baker et al., 2007; Riquelme and Rios, 2010; Teo et al., 2012). For an example, Li et al. (2019) reported the positive effect of gender, age, and income level on proenvironmental behavior or green consumption. Shao et al. (2018) found that people with higher income levels are more likely to pay for environmental protection. On the other hand, Du et al. (2018) reported a significant and negative impact of the level of income on green consumption. Song et al. (2019) found that education and income had no impact on green consumption. According to Wang et al. (2018), a higher education degree does not lead to a greater willingness to pay for green consumption; however, age can lead to a greater willingness to pay for green consumption. Then, related to the relationship between demographic factors and subjective norms, Venkatesh and Morris (2000) reported that females tend to be influenced by subjective norms compared to males. Riquelme and Rios (2010) concluded that gender plays a role in moderating the effect on adopting m-banking services through subjective norms in Singapore. However, Teo et al. (2012) fail to prove that gender has a significant positive association with subjective norms. Morris et al. (2005) reported that gender and age were significant moderators of the subjective norm on behavioral intention. In contrast, White Baker et al. (2007) fail to prove it. According to the above discussion, this study proposed the eight hypotheses to clarify how demographic factors will influence subjective norms rather than green purchasing decisions since subjective norms themselves will affect the purchasing decisions (see hypothesis 3).

H8: Demographic factors are positively affecting the subjective norms.

### Purchasing Intention, Purchasing Behavior, and Green Marketing

Referring to TRA or TPB, intentions and behaviors are significantly related when assessed at a similar level of specificity and when time differences between intention and behavior are concise (Ajzen and Fishbein, 2005). A high relationship between intentions and behavior can be seen in studies conducted by Wu and Chen (2014), Nguyen et al. (2019). However, the relations between intentions and behavior could vary, that many studies found no relationship between two constructs, or many studies observed inconsistency of the relationship. Consumers who declare their favorable views and intentions to engage in a proenvironmental manner do not transform their words into actions (Echegaray and Hansstein, 2017). This discrepancy is known as the green attitude-behavior gap (Park and Lin, 2018), the green intention-behavior gap (Frank and Brock, 2018), or the motivation-behavior gap (Frank and Brock, 2018). Several research initiatives are now focused on elucidating, comprehending, and resolving this issue. As a result of this condition, the ninth hypothesis proposed in this study aims to elucidate this occurrence.

H9: Purchase intention is positively affecting the purchase behavior for a green personal care product.

Kotler and Amstrong (2016) said that actual purchase behavior or purchase decision is a point in the buying process when customers finally purchase. The positive relationship between green marketing and purchase decision has been observed by Azimi and Shabani (2016), Sugoto et al. (2017), Dwipamurti et al. (2018), and Genoveva and Levina (2019). In addition, green marketing also increased in repurchase decisions. Since purchase intention is positively related to purchase behavior (hypothesis 9) and green marketing is also positively related to purchase behavior, this study extends the effect of green marketing on purchase behavior by placing the green marketing factor as a moderating variable. Thus, hypothesis 10 is proposed.

H10: Green marketing will strengthen the positive effect of purchase intention on purchase behavior for a green personal care product.

**FIGURE 1:**
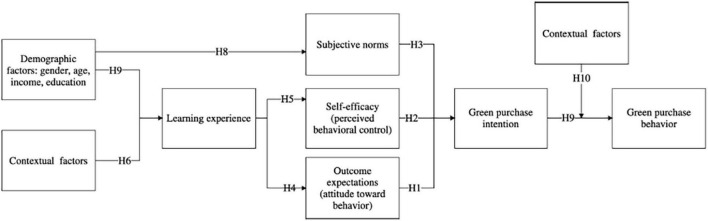
Conceptual model.

Finally, based on hypothesis 1 until hypothesis 10, the conceptual model of this study can be seen in Figure 1.

## Method of Research

### Variable and Measurement Items

In total, 42 items were used in this study. In detail, all items used in this study can be seen in Table 1. This research used a Likert scale with five categories (1 = strongly disagree until 5 = strongly agree) to measure the condition of all items, except demographic factors.

**TABLE 1:** Measurement items.

Factors (constructs)	Measurement items
Outcome expectation (adapted from Lin and Hsu, 2015; Nguyen et al., 2019) (GOE)	I think that practicing green consumer behavior by using green personal care product is conducive to sustainable development (GOE1)
	I think that practicing green consumer behavior by using green personal care product is respectful and promotes equality in terms of the recent ecological situation (GOE2)
	I think that I will only purchase personal care product if I know the origin (the manufacturer that produces them) (GOE3)
	I think the packaging and ingredient of green personal care product have a significant effect on reducing water and land pollution (GOE4)
	I think my consumption of green personal care product is conducive to provide a high-quality living environment (GOE5)
Self-efficacy (adapted from Paul et al., 2016) (GSE)	There are likely to be plenty of opportunities for me to purchase green products (GSE1)
	If it were entirely up to me, I am confident that I will purchase green products. (GSE2)
	I believe I can purchase green products (GSE3)
	I have the resources, time, and willingness to purchase green products (GSE4)
	I feel that purchasing green products is not totally within my control (GSE5)
Subjective norms (adapted from Paul et al., 2016) (GVB)	Most of the people who are important to me think that I should purchase green personal care products when going to purchasing (GVB1)
	Most people who are important to me would want me to purchase green products when going for purchasing (GVB2)
	People whose opinions I value would prefer that I purchase green personal care products (GVB3)
	My friend’s positive opinion influences me to purchase green personal care products (GVB4)
Learning experience (adapted and compressed from Böhlmark and Jinlei, 2020) (LE)	The outcome of my experience helped me to understand the environmental issues (LE1)
	The outcome of my experience helped me understand the negative impact of personal care product on the environment (LE2)
	The outcome of my experience helped me able to learn from the concrete example that I could to relate to reduce the negative environmental impact from personal care products (LE3)
	The outcome of my experience helped me to understand how using the personal care product is giving a negative impact on the environment (LE4)
	The outcome of my experience helped me to understand what I was expected from using green personal care products (LE5)
Contextual factors (adapted from Joshi and Rahman, 2015) (CF)	The green personal care products are available in sufficient quantities in supermarkets (CF1)
	Green personal care products can be found easily among several similar products (CF2)
	The green personal care products sold at a low or reasonable price (CF3)
	Green personal care products produced by a brand that has a good image (CF4)
	The green personal care products are labeled with eco-labeling, or eco-certification informs consumers about the green characteristics of the product (CF5)
Green purchase intention (adapted from Nguyen et al., 2019) (GI)	I will consider buying green personal care product because they are less polluting (GI1)
	I plan to switch to another brand for ecological reasons (GI2)
	I plan to pay more for a green personal care product that helps protect the environment (GI3)
	I plan to purchase green personal care in the next month (GI4)
Green purchase behavior (adapted from Nguyen et al., 2019) (GPB)	I prefer purchasing safe or traceability personal care product (GPB1)
	I prefer purchasing personal care product with the green label (GPB2)
	I rarely use personal care product with non-recycled packaging (GPB3)
	Personal care product using by my family are green product (GPB4)
	I introduce the green personal care product I use to my relatives and friends (GPB5).
Green marketing (adapted from do Paco et al., 2019) (GM)	Green advertising is a necessary form of advertising of personal care products (GM1)
	I tend to pay attention to the green advertising message, especially for personal care (GM2)
	I respond favorably to brands of personal care products that use green messages in their advertising (GM3)
Factors (constructs)	Measurement items
	I am the kind of customer who is willing to purchase personal care products marketed as being green (GM4)
	The use of green messages in advertising of personal care products affects my attitude toward the advertising (GM5)

### Data Collection Procedure

For this study, Google Forms was utilized to develop and produce web-based surveys. Then, this study collects data from web-based surveys through a combination of a non-probability of convenience and purposive sampling technique. This study prefers to choose participants with age older than 17 years because it is considered to have the ability to make purchasing decisions. Then, the participants were recruited by sending a copy of the URL of the web-based surveys *via* email or other social media.

### Data Processing Technique

For data processing, this study employed partial least squares (PLS) through the SmartPLS 3.0 software that manufactured at Germany. PLS is a statistical approach that depends on variance measurement, which has two advantages. First, we may apply PLS without making any assumptions about the distribution of the data (Vinzi et al., 2010). PLS requires no normal data and may be utilized with categorical or ordinal (quasi-metric) data (Hair et al., 2014). The second advantage is that PLS may be used to tiny quantities of data (Wong, 2013).

## Results

### Profile of Respondents

After removed outlier data that did not meet the criterion (e.g., dishonest answers or lacking values), this study succeeded in obtaining 602 valid data from filling out questionnaires by participants. In detail, the profile of respondents can be seen in Table 2.

**TABLE 2:** Demographic profile of the participant.

Demographic factors	Categories	Frequency	Percentage
Gender (DF1)	Male	303	50.30%
	Female	299	49.70%
Age (DF2)	Less than 26 years	125	20.76%
	26- less than 35	201	33.39%
	36- less than 45	105	17.44%
	45- less than 56	141	23.42%
	above 56 years	28	4.65%
Level of education (DF3)	Senior high school or diploma I or lower	110	18.30%
	Diploma III	60	10.00%
	Diploma IV or bachelor	326	54.10%
	Master	87	14.50%
	Doctoral degree or hinger	19	3.20%
Level of income (DF4)	less than USD 133.33	86	14.30%
	USD 133.33–less than 333.33 USD	185	30.70%
	USD 333.33–less than 666.66 USD	189	31.40%
	above 666.66 USD	142	23.60%

### Path Diagram

**FIGURE 2:**
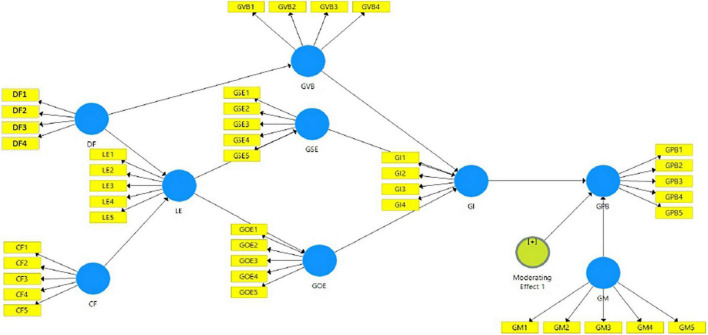
Path diagram of conceptual model.

The path diagram of the conceptual model can be seen in Figure 2.

### Result of Evaluation of Measurement Models

To test the validity and reliability of the measurement models for each factor (construct), the reflective measurement models were evaluated through convergent and discriminant validity and reliability test. To guarantee convergent validity, all items with factor loadings < 0.5 were excluded from further analysis (Fornell and Larcker, 1981). Following that, the factor loading of each item is recalculated, as are the values of average variance extracted (AVE), composite reliability (CR), and Cronbach’s alpha value of each factor (construct). Table 3 shows the initial and final factor loadings of each item, as well as the values of AVE, CR, and Cronbach’s alpha value of each factor (construct). Table 3 shows eight items, namely, GOE1, GV3, LE2, CF1, CF3, GI2, GM1, DF1, and DF4, that were eliminated from further analysis.

**TABLE 3:** The initial and final factor loading of each item and the value of AVE, CR and Cronbach’s α of each factor (construct).

Factors (constructs)	Measurement items	Mean	Sdt.Dev	First factor loading	Final factor loading	AVE	CR	Cronbach’s Alpha	
Outcome expectation (GOE)	GOE1	3.940	1.092	0.515	-	0.622	0.868	0.801	
	GOE2	4.591	0.679	0.730**	0.742**				
	GOE3	4.648	0.631	0.756**	0.785**				
	GOE4	4.282	0.813	0.815**	0.812**				
	GOE5	4.316	0.771	0.795**	0.813**				
Self-efficacy (GSE)	GSE1	*4.252*	*0.775*	0.850**	0.852**	0.635	0.896	0.855	
	GSE2	*4.163*	0.831	0.864**	0.865**				
	GSE3	*3.975*	0.901	0.759**	0.757**				
	GSE4	*4.339*	0.763	0.827**	0.827**				
	GSE5	*3.970*	0.924	0.667*	0.665*				
Subjective norms (GVB)	GVB1	3.909	0.931	0.767**	0.799**	0.698	0.874	0.786	
	GVB2	3.580	0.982	0.841**	0.875**				
	GVB3	3.535	1.104	0.550	-				
	GVB4	3.296	1.061	0.838**	0.831**				
Learning experience (LE)	LE1	4.716	0.594	0.709**	0.725**	0.666	0.888	0.831	
	LE2	4.178	1.078	0.497	-				
	LE3	4.414	0.729	0.813**	0.807**				
	LE4	4.334	0.839	0.831**	0.846**				
	LE5	4.331	0.775	0.864**	0.879**				
Contextual factors (CF)	CF1	3.819	1.095	0.578		0.573	0.800	0.647	
	CF2	3.638	1.064	0.709**	0.679*				
	CF3	3.332	1.037	0.523					
	CF4	4.003	0.893	0.736**	0.775**				
	CF5	3.776	0.969	0.789**	0.811**				
Green purchase intention (GI)	GI1	4.229	0.743	0.820**	0.841**	0.767	0.908	0.848	
	GI2	3.970	0.872	0.546	-				
					loading			Alpha	
	GI3	3.895	0.840	0.877**	0.880**				
	GI4	4.032	0.801	0.883**	0.905**				
Green purchase behavior (GPB)		GPB1	4.038	0.756	0.781**	0.78**	0.540	0.854	0.787
		GPB2	3.573	0.900	0.795**	0.796**			
	GPB3	3.887	0.965	0.680*	0.678*				
	GPB4	3.256	0.965	0.666*	0.668*				
	GPB5	3.880	0.894	0.741**	0.742**				
Green marketing (GM)	GM1	3.945	0.868	0.594		-	0.529	0.817	0.708
	GM2	3.997	0.801	0.701**	0.700**				
	GM3	4.355	0.729	0.676*	0.675*				
	GM4	3.703	0.983	0.803**	0.803**				
	GM5	3.779	0.988	0.723**	0.723**				
Demographic (DF)	DF1 (age)			0.235		0.697	1.000	0.599	
	DF2 (gender)			0.763**	0.935**				
	DF3 (education			0.712**	0.721**				
		DF4			0.133	-			
		(income)							

*Valid factor loading > 0.6; **strong factor loading > 0.7.

This study supports the construct if the AVE > 0.5 (Fornell and Larcker, 1981), the CR > 0.6 (Fornell and Larcker, 1981), and the Cronbach’s alpha > 0.6 (Akter et al., 2011). As seen in Table 3, all constructs have AVE > 0.5, and all constructs have CR and Cronbach’s alpha > 0.6. As a consequence, based on the final factor loading of all items and the values of AVE, CR, and Cronbach’s alpha values for all constructs, the convergent validities of all items are sufficient, and the calculation model also demonstrates that each construct displayed appropriate reliability. Then, Table 4 shows the discriminant validity result from the final iteration. Evaluating the factor loading inside the columns in Table 4 reveals that in all circumstances, the factor loading of an item within its construct is larger than any of its cross-loadings with other constructs.

**TABLE 4:** The result of discriminant validity.

Factors (construct)	Indicator	CF	DF	GI	GM	GOE	GPB	GSE	GVB	LE
Contextual factors (CF)	CF2	0.679*	–0.026	0.191	0.309	0.095	0.259	0.24	0.357	0.156
	CF4	0.775*	0.081	0.286	0.297	0.301	0.259	0.361	0.267	0.321
	CF5	0.811*	–0.019	0.263	0.373	0.256	0.334	0.340	0.317	0.284
Demographic factor	DF2	0.043	0.935*	0.161	0.136	0.114	0.09	0.144	0.131	0.159
	DF3	–0.014	0.721*	0.064	0.035	0.104	0.069	0.093	–0.059	0.137
Green purchase intention (GI)	GI1	0.290	0.090	0.841*	0.581	0.532	0.575	0.651	0.301	0.559
	GI3	0.293	0.132	0.880*	0.572	0.452	0.676	0.616	0.356	0.454
	GI4	0.298	0.165	0.905*	0.599	0.473	0.669	0.643	0.395	0.500
Green marketing (GM)	GM2	0.430	0.030	0.393	0.700*	0.344	0.431	0.444	0.340	0.332
	GM3	0.261	0.057	0.494	0.675*	0.54	0.404	0.490	0.219	0.472
	GM4	0.273	0.103	0.597	0.803*	0.326	0.656	0.576	0.331	0.307
	GM5	0.310	0.148	0.423	0.723*	0.26	0.438	0.381	0.430	0.263
Outcome expectation (GOE)	GOE2	0.208	0.041	0.283	0.265	0.742*	0.209	0.343	0.059	0.497
	GOE3	0.280	0.049	0.371	0.353	0.785*	0.335	0.475	0.175	0.501
	GOE4	0.242	0.159	0.533	0.43	0.812*	0.410	0.521	0.271	0.597
	GOE5	0.261	0.128	0.506	0.461	0.813*	0.430	0.493	0.241	0.580
Green purchase behavior (GPB)	GPB1	0.282	0.104	0.637	0.503	0.431	0.780*	0.576	0.251	0.447
	GPB2	0.223	0.137	0.612	0.586	0.294	0.796*	0.526	0.311	0.299
	GPB3	0.320	–0.02	0.407	0.461	0.290	0.678*	0.420	0.215	0.304
	GPB4	0.237	0.122	0.402	0.418	0.162	0.668*	0.356	0.276	0.222
	GPB5	0.324	0.000	0.575	0.528	0.438	0.742*	0.505	0.403	0.409
Self-efficacy (GSE)	GSE1	0.338	0.088	0.651	0.549	0.524	0.573	0.852*	0.334	0.581
	GSE2	0.327	0.158	0.663	0.555	0.487	0.565	0.865*	0.355	0.505
	GSE3	0.363	0.126	0.536	0.545	0.385	0.532	0.757*	0.356	0.357
	GSE4	0.324	0.141	0.561	0.471	0.556	0.487	0.827*	0.320	0.608
	GSE5	0.387	0.064	0.456	0.533	0.357	0.477	0.665*	0.395	0.321
Subjective norms (GVB)	GVB1	0.355	0.027	0.284	0.324	0.247	0.271	0.338	0.799*	0.270
	GVB2	0.306	0.094	0.335	0.363	0.171	0.303	0.377	0.875*	0.172
	GVB4	0.337	0.067	0.377	0.432	0.215	0.409	0.367	0.831*	0.226
Learning Experience (LE)	LE1	0.279	0.056	0.370	0.313	0.56	0.312	0.422	0.127	0.725*
	LE3	0.279	0.187	0.507	0.430	0.549	0.397	0.529	0.206	0.807*
	LE4	0.306	0.157	0.424	0.327	0.545	0.355	0.462	0.231	0.846*
	LE5	0.297	0.166	0.557	0.416	0.61	0.445	0.571	0.281	0.879*

*Indicated that the item belong to certain construct.

### Result of Evaluation of Structural Model

The validity of the structural model used in this study is discussed in the following subsections. In this case, this study uses the coefficient of determination (R^2^), Q2, f2, goodness of fit (GoF) index, the χ^2^/degree of freedom, standardized root means square residual (SRMR), and the normed fit index (NFI) for assessing the validity of the structural model. The result can be seen in Table 5.

**TABLE 5:** The R^2^ value, Q^2^ value, f^2^ value, GoF index, SRMR, the χ2/degree of freedom, SRMR, and NFI for a hypothesized model.

Statistical test		Value	Cut-off value	Result
R^2^	R^2^ GI	0.561	0.19-weak; 0.33- moderate;0.67- strong/substantial^a^	Moderate
	R^2^ GOE	0.482		Moderate
	R^2^ GSE	0.610		Moderate
	R^2^ GPB	0.374		Moderate
	R^2^ GVB	0.019		Weak
	R^2^ LE	0.154		Weak
Q^2^		0.945	Q^2^ > 0^b^	d predictive relevance- close to 1
f^2^	GOE GI; GSEGI	0.055; 0.414	0.02-weak;0.15-moderate; 0.35-strong^c^	Weak; Strong
	LE GOE	0.931		Strong
	LEGSE	0.598		Strong
	GI GPB; GM GPB	0.362; 0.170		Strong; Moderate
	DF GVB	0.019		Weak
	CF LE; DFLE	0.143; 0.024		Weak (close to moderate);
GoF		0.395	0.1-small; 0.25-moderate; and 0.36-large^c^	Weak
				Large
SRMR		0.077	Less than 0.08 -good fit; 0.05- 0.1-an adequate fit^d^	Good Fit
c^2^/df		2.400	0.00–2.00: good mode; up to 3.00 a reasonable fit^e^	Reasonable fit
NFI		0.703	Higher than 0.90 -a good fit; 0.50 to less than 0.80- marginal fit ^e,f^	Marginal fit

*Source: ^a^Chin (1998); ^b^Vinzi et al. (2010); ^c^Tenenhaus et al. (2005); ^d^Hu and Bentler (1999), Senel (2011), and Dede and Ayranci (2014); ^e^Schermelleh-Engel et al. (2003), Holmes-Smith (2000); ^f^Ghozali (2011).*

### Result of Hypothesis Testing

The hypothesis test results are shown in Table 6. If a *p*-value < 0.05 exists between the independent and dependent variables, the hypothesis is accepted.

**TABLE 6:** Result of hypothesis testing.

	Relationship			Factor Loading (b)	*t*-value	*p*-value	Result
H1	Outcome expectation	→	Purchase intention for green personal care product	0.193	8.564	(0.000)	Accepted
H2	Self-efficacy	→	Purchase intention for green personal car product	0.566	13.692	(0.000)	Accepted
H3	Subjective norms	→	Purchase intention for green personal care product	0.108	3.347	(0.001)	Accepted
H4	Learning experiences	→	Outcome expectation	0.694	22.835	(0.000)	Accepted
H5	Learning experiences	→	Self-efficacy	0.612	16.140	(0.000)	Accepted
H6	Contextual factors	→	Learning experiences	0.350	10.418	(0.000)	Accepted
H7	Demographic factors	→	Learning experiences	0.144	0.058	(0.013)	Accepted
H8	Demographic factors	→	Subjective norms	0.135	2.092	(0.036)	Accepted
H9	Purchase intention	→	Purchase behavior	0.510	11.559	(0.000)	Accepted
H10	Purchase Intention	→	Purchase				Accepted
		Green Marketing	Behavior	0.064	2.096	(0.036)	Accepteds

Based on Table 6, outcome expectation (β = 0.193, *p* < 0.05), self-efficacy (β = 0.566, *p* < 0.05), and subjective norms. (β = 0.108, *p* < 0.05), all positively affected purchase intention for green personal care product. Hence, H1, H2, and H3 were all supported. Learning experience affected outcome expectation (β = 0.694, *p* < 0.05) and self-efficacy (β = 0.612, *p* < 0.05). H4 and H5 were supported. Contextual factors affected learning experiences (β = 0.350, *p* < 0.05), whereas demographic factors affected learning experiences (β = 0.144, *p* < 0.05) and subjective norms (β = 0.135, *p* < 0.05). Thus, H6, H7, and H8 were supported. Finally, purchase intention affected purchase behavior (β = 0.510, *p* < 0.05) and green marketing moderate intention to actual purchase of green personal care product relation. Hence, H9 and H10 were also supported.

## Conclusion

A recent study adds to the broader literature on green purchasing behavior. This study suggests that self-efficacy, outcome expectation, and subjective norms play a vital role in influencing purchase intention for green personal care products. One of the startling findings in this study was that self-efficacy and outcome expectation more influencing the purchase intention of green personal care than subjective norms. This can be attributed to the fact that initiating from oneself over the external factors was more dominant for purchasing intention of personal care products. Then, developing a learning experience was an important part of encouraging the self-efficacy and outcome expectation of customers, and that contextual factors influenced learning experience in developing consumption-related attitudes to green personal care products. Moreover, although the behavior of consumers to purchase green personal care products is highly influenced by their intention, green marketing has an essential role in strengthening the relationship.

The results that arisen from this study propose the theoretical and managerial implications. In theoretical implications, first, the research may be helpful to those studying the behavior of individuals and, in particular, customer behavior as it leads to enhancing science literature relevant to human choice factors. Then, since all of the proposed hypotheses were fulfilled, this condition highlighted the potential of variables to build customer choices profoundly. Precisely, starting from the consideration of the modification of classical variables adopted in the TPB framework through including the variable from SCT, SLT, and green marketing, the analysis confirmed the incidence of outcome expectation, self-efficacy, subjective norms, contextual factors, and learning experiences on the behavioral intention of people, which, in turn, was able to affect the actual behavior in purchasing the green personal care product. This study confirmed the theoretical framework of Ajzen (1991) similar to numerous other literary studies. However, introducing three other variables (i.e., learning experience, contextual factors, and green marketing) to extend the TPB framework, it is highlighted that simply considering the classical variables of TPB could be insufficient, at least in forcing the green customer to purchase the TPB green customer care product. In fact, in deciding to purchase the green product, the behavioral intention and actual behavior are induced by situational factors as well as green advertising from the company and those conditions suggest to scholars the importance of not being restricted to the application of the TPB for the investigation of the phenomena conditioning the choice of green product but to propose based on what has been shown by prior findings, including new and broader conceptualizations.

The proposed model and its findings will provide empirical proof of the causes or variables that influence customer behavioral intentions to buy green personal care products in managerial implication. In particular, this work can be considered beneficial to making decisions that can be used by entrepreneurs and managers who need to understand customer preferences and the explanations for such buying decisions, especially in green personal care. Understanding why people behave the way they do helps firms to predict potential patterns, giving them more time to identify and execute plans that can meet their demands and, as a result, retain them. In this respect, the study stressed that the decision of consumers to use a green personal care product was not based on chance but rather on easily detectable factors and, therefore, manageable. For example, the management and entrepreneur should pay attention to self-efficacy since this factor has been proven to influence the behavioral intention of an individual. The customer seems to choose what they believe they can manage if they have the requisite resources. In other words, it may be helpful to make consumers believe that they have all of the resources necessary to obtain a personal care product that adheres to green practices. Since companies generally charge a premium for green products while consumers are usually sensitive toward price (they are willing to buy green products but not at higher prices), those who manage a green personal care product should focus on efforts to reduce the prices of the product following the willingness to pay from the customer who sensitizes to price (it assumes that the willingness to pay of customers has been accorded to ownership of resources by the consumer). The companies should overthink the “pricing strategies” that make the product a “niche product” consumable only by a section of society rather than a mass product that everyone can consume. It could be said that pricing is one solution to make customers believe that they have sufficient resources to buy the green personal care product. The other manager or entrepreneurship can attract customers who have limited time and do not like to search for environmentally sustainable products. Those who manage a green personal care product should focus on easily accessible/available green personal care products in the supermarket.

Another managerial implication related to the positive effect of outcome expectation on green purchase intention and the role of green marketing to moderate the relationship between intention to actual purchase behavior suggests that those who manage a green personal care product should focus on giving information to customer related to the effect of their consumption patterns on nature and society. This information boost the buy intention and real purchase motivation of customers by allowing them to objectively analyze the benefits of green purchasing activities and contemplate how their actions might help nature and society. In addition to providing the information, policymakers can cultivate and further develop it through environmental education, and marketers can conduct campaigns to increase public awareness of green personal care products, inform consumers of the meaning and availability of green personal care products, and proclaim the advantages of using green personal care products.

There are limitations to this study, just like any other. In selecting articles for this review, the authors tried to be both systematic and accurate, but there are still some shortcomings that could be addressed in future studies. Individuals from different cultures and social backgrounds may experience different effects from the variables identified. This study considered the impact of demographic factors but did not separately test each demographic factor in the conceptual model. Future studies may explore this limitation by testing the conceptual model for specific demographic factors and compared the result obtained. The other limitation of the study is related to the use of questionnaires for data collection. Even though using a questionnaire as quantitative analysis has advantages in terms of sample size and accessibility, it did not allow us understand why the customer selects green personal care products. To solve this limitation, the additional study could be conducted in the future by using qualitative analysis (e.g., detailed interviews) and compared the findings whether it is identical to the ones produced in this quantitative analysis.

## Data Availability Statement

The original contributions presented in the study are included in the article/supplementary material, further inquiries can be directed to the corresponding author/s.

## Ethics Statement

Ethical review and approval was not required for the study on human participants in accordance with the local legislation and institutional requirements. Written informed consent from the participants was not required to participate in this study in accordance with the national legislation and the institutional requirements.

## Author Contributions

AS and NP conceived and designed the study. PL participated in the acquisition of data. NP analyzes the data. AS and BT gave advice on methodology and drafted the manuscript. AS and PL revised the manuscript. AS is the guarantor of this work and had full access to all the data in the study and takes responsibility for its integrity and the accuracy of the data analysis. All authors read and approved the final manuscript.

## Conflict of Interest

The authors declare that the research was conducted in the absence of any commercial or financial relationships that could be construed as a potential conflict of interest.

## Publisher’s Note

All claims expressed in this article are solely those of the authors and do not necessarily represent those of their affiliated organizations, or those of the publisher, the editors and the reviewers. Any product that may be evaluated in this article, or claim that may be made by its manufacturer, is not guaranteed or endorsed by the publisher.
